# Application of codon usage and context analysis in genes up- or down-regulated in neurodegeneration and cancer to combat comorbidities

**DOI:** 10.3389/fnmol.2023.1200523

**Published:** 2023-06-13

**Authors:** Rekha Khandia, Megha Katare Pandey, Magdi E. A. Zaki, Sami A. Al-Hussain, Igor Baklanov, Pankaj Gurjar

**Affiliations:** ^1^Department of Biochemistry and Genetics, Barkatullah University, Bhopal, Madhya Pradesh, India; ^2^Translational Medicine Center, All India Institute of Medical Sciences, Bhopal, India; ^3^Department of Chemistry, Imam Mohammad Ibn Saud Islamic University (IMSIU), Riyadh, Saudi Arabia; ^4^Department of Philosophy, North Caucasus Federal University, Stavropol, Russia; ^5^Department of Science and Engineering, Novel Global Community Educational Foundation, Hebersham, NSW, Australia

**Keywords:** codon usage, codon pattern, synonymous codons, neurodegeneration, cancer, CRISPR/Cas

## Abstract

**Introduction:**

Neurodegeneration and cancer present in comorbidities with inverse effects due to the expression of genes and pathways acting in opposition. Identifying and studying the genes simultaneously up or downregulated during morbidities helps curb both ailments together.

**Methods:**

This study examines four genes. Three of these (Amyloid Beta Precursor Protein (*APP*), Cyclin D1 (*CCND1*), and Cyclin E2 (*CCNE2*) are upregulated, and one protein phosphatase 2 phosphatase activator (PTPA) is simultaneously downregulated in both disorders. We investigated molecular patterns, codon usage, codon usage bias, nucleotide bias in the third codon position, preferred codons, preferred codon pairs, rare codons, and codon context.

**Results:**

Parity analysis revealed that T is preferred over A, and G is preferred over C in the third codon position, suggesting composition plays no role in nucleotide bias in both the upregulated and downregulated gene sets and that mutational forces are stronger in upregulated gene sets than in downregulated ones. Transcript length influenced the overall %A composition and codon bias, and the codon AGG exerted the strongest influence on codon usage in both the upregulated and downregulated gene sets. Codons ending in G/C were preferred for 16 amino acids, and glutamic acid-, aspartic acid-, leucine-, valine-, and phenylalanine-initiated codon pairs were preferred in all genes. Codons CTA (Leu), GTA (Val), CAA (Gln), and CGT (Arg) were underrepresented in all examined genes.

**Discussion:**

Using advanced gene editing tools such as CRISPR/Cas or any other gene augmentation technique, these recoded genes may be introduced into the human body to optimize gene expression levels to augment neurodegeneration and cancer therapeutic regimens simultaneously.

## 1. Introduction

Cancer promotes continuous proliferation, invasion, and metastasis of malignant cells into distal organs. In contrast, neurodegeneration is characterized by neuronal dysfunction and death. These disorders display several opposite features. Where cancer is characterized by abnormal cell survival and resistance to cell death, cells in neurodegenerative disease are at elevated risk of cell death. Inverse comorbidities have been reported in cancer and neurodegeneration in several reports (Ferreira et al., 2010; Driver et al., 2012; Driver, 2014). Transcriptomic meta-analyses have investigated inverse comorbidities in terms of molecular processes common to CNS disorders and cancers. A significant overlap has been reported between genes that are up-regulated in cancer and down-regulated in neurodegeneration, and vice versa (Ibáñez et al., 2014). Inverse comorbidities are common. Thus, genes and pathways regulated in opposite directions have been thoroughly investigated and understood, and examples of such genes and pathways are available. To date, only a few reports describe pathways operating in same direction in cancer and neurodegeneration. We thus investigated genes implicated in both ailments, to identify ways to simultaneously address cancer and neurodegeneration. We found many genes to be present at the interface of cancer and neurodegeneration, including α-synuclein, *PINK1*, *DJ-1*, *LRRK2*, *ATP13A2*, *PLA2G6*, *MAPT*, and *CDK5* (Plun-Favreau et al., 2010) with disease-associated point mutations at various sites (Mavrou et al., 2008; Morris et al., 2010; Veeriah et al., 2010a,b). Specific genes and pathways that simultaneously increased CNS disorder risk while reducing that of cancer were identified. Transcriptomic meta-analyses revealed the simultaneous upregulation of 74 genes, for example *PPIAP11*, *IARS*, *GGCT*, *NME2*, *GAPDHP1*, *CDC123*, *PSMD8*, *MRPS33*, *FIBP*, and *OAZ2* in three CNS disorders and downregulation in three cancer types (Ibáñez et al., 2014). Similarly, 19 genes were up-regulated in three cancer types (lung, prostate, and colorectal), and down-regulated in three CNS disorders (Alzheimer’s disease, Parkinson’s disease and Schizophrenia) and the examples are *MT2A*, *MT1X*, *NFKBIA*, *AC009469.1*, *DHRS3*, *CDKN1A*, and *TNFRSF1A* (Ibáñez et al., 2014). In cancer, *P53* is down-regulated, whereas *PIN* and Cyclin F are up-regulated. At the same time, *P53* is up-regulated, while *PIN* and Cyclin F are down-regulated in neurodegeneration. Inverse comorbidities make coupled treatment of both diseases difficult.

To find a solution for both diseases, we looked for genes that were up-regulated or down-regulated simultaneously in both disorders, so that they could be handled together. An extensive literature search led us to four genes, amyloid precursor protein (*APP*), Cyclin D, Cyclin E, and protein phosphatase 2A (*PP2A/PTPA*). In cancer and neurodegeneration, *APP*, Cyclin D, and Cyclin E are up-regulated, whereas *PTPA* is down-regulated.

Chromosome 21 trisomy, the presence of *APP* on chromosome 21, and association of *APP* gene upregulation with increased risk of hematologic malignancy in patients with Down syndrome (DS) suggest that *APP* might predispose to cancer. Children with Down syndrome are at 10- to 20-fold higher risk of acute lymphoblastic leukemia and acute myeloid leukemia. In patients with acute myeloid leukemia, *APP* is most overexpressed (Wang et al., 2010), and its overexpression is associated with poor prognosis in oral squamous cell carcinoma (Lin et al., 2020). *APP* overexpression in mouse models leads to neuronal death (Cheng et al., 2016). Overexpression of the human *APP* gene in *Drosophila melanogaster* results in cholinergic and dopaminergic brain neurons that are significantly degenerated later in life compared with controls, accompanied by memory deficits and poor cognitive abilities (Bolshakova et al., 2014).

Cyclins D and E have been reported to be up-regulated, whereas *PTPA* has been reported to be down-regulated in cancer and neurodegenerative disease [reviewed in (Seo and Park, 2020)]. Cyclins control the cell cycle by modulating Cyclin-dependent kinases (CDKs), and their dysregulation underlies several human cancers (Krasniqi et al., 2022; Wu et al., 2022; Sher et al., 2023). In addition to cell cycle regulation, Cyclins participate in cellular processes specific to terminally differentiated neurons (Zhou and Ekström, 2022).

Cyclins play important roles in neuronal physiology and pathology (Cho et al., 2015). Cyclin D1 is a regulatory subunit of *CDK4* or *CDK6* and is essential for entry into S phase from G1. Mutations leading to aberrant overexpression of Cyclin D alter cell cycle progression and may contribute to tumorigenesis. Thus, *CCND1* overexpression correlates with shorter survival and poorly differentiated gastric cancer and other tumors (Shan et al., 2017). Cyclin D1 is associated with apoptosis in post-mitotic neurons (Shupp et al., 2017). In a study of 117 subjects, Cyclin D levels were significantly higher in patients with Alzheimer’s disease (AD; Kim et al., 2016). *CDK4* induces the re-entry of neurons into the cell cycle, is deleterious to terminally differentiated neurons, and may lead to neuronal degeneration (McShea et al., 1997). Cyclin D1 is involved in breast cancer cell invasion/migration, and its overexpression increases invasion (Gao et al., 2020). Cyclin E is a regulatory subunit of *CDK2* that initiates DNA replication during G1/S transition. Its overexpression, resulting in genomic instability, has been reported in triple-negative breast cancer (Chen et al., 2018), non-Hodgkin’s lymphoma (Williams and Swerdlow, 1994), lung cancer (Eymin and Gazzeri, 2010), pancreatic cancer (Pang et al., 2020), and liver cancer (Sonntag et al., 2021) and results in genomic instability (Kok et al., 2020). Increased Cyclin D and E levels are evident in degenerating neurons exposed to the neurotoxin 1-methyl-4-phenylpyridinium (Höglinger et al., 2007). Elevated Cyclin E levels are observed during spinal cord injury which induce cell cycle activation and neuronal apoptosis (Tian et al., 2006).

Phosphotyrosyl phosphatase activator (*PTPA*/*PP2A*), a member of the serine/threonine protein phosphatase family, is a tumor suppressor gene product. Its inactivation has been reported in endometrial carcinomas (Remmerie and Janssens, 2019). This inactivation induces cell transformation (Sablina et al., 2010). *PTPA* is decreased in the brains of Alzheimer’s disease (AD) mouse models. Additionally, *PTPA* is present in the mitochondrial membrane, and its knockdown induces apoptosis in neuronal cell lines (Luo et al., 2014).

Relative synonymous codon usage (RSCU) explains bias in codon usage within genes or transcripts. This bias can result from various evolutionary (selection, mutation, and GC-biased gene conversion) and compositional factors. Codon usage impacts the level of gene expression through its effect on transcription (Zhou et al., 2016). Preferred codons are commonly present in highly expressed genes, whereas poorly expressed genes contain rare or less common codons. Rare codons in *Escherichia coli*, including AGG, AGA, CUA, AUA, CGA, and CCC, regulate different endogenous proteins. Expression is limited due to the rarity of their cognate tRNAs (Wang et al., 2016). When RNA polymerase encounters rare codons, transcription generally pauses, resulting in ribosome disassembly (Rosano and Ceccarelli, 2009). Rare codons are generally found in nonrandom clusters (Clarke and Clark, 2008). Codon pair bias is a variant form of codon bias, and is the probability of the presence of two specific adjacent codons. For example, for the adjacent amino acids alanine and glutamate, there are eight possible codon pairs, and all should be equally present; however, the GCC-GAA pair is highly underrepresented despite containing GCC, the most prevalent codon encoding alanine (Coleman et al., 2008).

Codon bias may be applied as a tool in synthetic biology to create synthetic gene constructs capable of high level expression (Supek and Šmuc, 2010), to reduce expression when constructing attenuated vaccine candidates (Giménez-Roig et al., 2021), or to create new genomes (Tulloch et al., 2014). In the present study, we envisaged codon bias, its correlation with various molecular features of transcripts, expression profile, preferred and rare codons, codon pairs, and codon context for the genes *APP*, *Cyclin* D, and *Cyclin* E, which are up-regulated, and *PTPA*, which is down-regulated in both cancer and neurodegeneration. The information in this study will help modulate and fine-tune the expression of these genes, contributing to strategies for controlling these ailments concurrently.

## 2. Materials and methods

### 2.1. Sequence retrieval

All transcripts corresponding to the genes *APP* (11), *CCND1* (1), *CCNE1* (4), and *PTPA* (06) were retrieved from the National Center for Biotechnology (NCBI) GenBank database.^1^ Transcripts containing a reading frame starting with ATG and ending with a stop codon were included in this study. Accession numbers and transcript lengths are listed in Table 1.

**Table 1:** List of transcripts examined in this study corresponding to *APP*, *CCND1*, *CCNE1*, and *PTPA* genes.

S. No.	Name of gene	Accession number of transcript	Length of transcript
1	*APP*	NM_000484	2,313
		NM_201413	2,256
		NM_201414	2088
		NM_001136016	2,241
		NM_001136129	1920
		NM_001136131	1983
		NM_001204301	2,259
		NM_001204302	2,202
		NM_001385253	2,145
		NM_001136130	2,145
		NM_001204303	2034
2	*CCND1*	AF511593	888
13	*CCNE1*	NM_001322262	1,188
		NM_001322261	1,086
		NM_001322259	1,098
		NM_001238	1,233
17	*PTPA*	NM_178001	1,077
		NM_001193397	867
		NM_001271832	885
		NM_021131	972
		NM_178003	846
		NM_178000	972

### 2.2. Principal component analysis

Principal component analysis (PCA) is a multivariate tool used to determine major variation trends. PCA was performed using RSCU values to identify major codon usage trends in up-regulated and down-regulated genes. The up-regulated gene group consisted of transcripts encoded by *APP*, *CCND1*, and *CCNE1*, while the down-regulated gene group consisted of transcripts encoded by *PTPA*. A PCA plot was constructed using the first two axes, which accounted for maximum variation. The figure was made using Origin18 software.

### 2.3. Protein properties determination

Protein physical properties affect their biological behaviors and influence their codon usage. Various protein properties have been reported to correlate with nucleotide composition and codon bias (Khandia et al., 2021). In this study, we calculated two protein properties: GRAVY and AROMA. GRAVY assesses in combination both hydrophobicity and hydrophilicity, with GRAVY scores ranging between − 2 and + 2. Positive values suggest hydrophobicity and negative values indicate hydrophilicity. AROMA determines the frequency of aromatic amino acids (Phe, Tyr, and Trp) in a given protein (Alqahtani et al., 2022). These protein indices suggest the action of selective forces (Khandia et al., 2019). Both indices were calculated using COUSIN (COdon Usage Similarity INdex) software developed by Bourret et al. (2019).

### 2.4. Scaled Chi-square

Shields et al. (1988) suggested a term to quantitate bias based on a Chi-squared (*χ*^2^) value, called the scaled Chi-square (SCS). This SCS value is derived from the equal usage of codons from synonymous codon groups normalized to actual usage, with tryptophan and methionine excluded. SCS values range between 0 and 1, with higher values suggesting a higher bias (Bahiri-Elitzur and Tuller, 2021).

### 2.5. Codon adaptation index

The Codon Adaptation Index (CAI) was initially developed to determine codon bias in DNA and RNA sequences. It calculates the similarity in codon usage between a given gene and codon usage in highly expressed genes from a reference set (Puigbò et al., 2008). It also predicts gene expression level and is thus frequently used in heterologous gene expression (Raab et al., 2010). CAI is not comprehensive, but is an important measure for determining protein expression, and has been verified using deep learning methods and biological experiments (Fu et al., 2020). In the present study, the CAI values for each transcript were calculated and used for correlation studies.

### 2.6. Rare codon analyses

Rare codons occur at low frequencies in genes and transcripts. Rare codons transiently stall ribosomes, helping proteins fold properly (Li et al., 2006). Rare codon frequencies were derived and the frequency of rare codons was adjusted according to transcript length. Codons with a percentage occurrence below 0.5% were considered rare.

### 2.7. Codon context analysis

Codon context refers to the tendency of codons to be found in pairs. Generally, a few codon pairs are used more than others, and codon pair bias is present in organisms (Kunec and Osterrieder, 2016). Codon pair bias has been implicated in reducing protein expression via codon pair de-optimization while generating attenuated vaccine candidates using a synthetic biology approach (Coleman et al., 2008). Therefore, the codon pair context was derived and analyzed for all four genes in this study.

### 2.8. Effective number of codons

Effective number of codons (ENc) is a metric in which bias is measured in terms of deviation from random distribution of synonymous codons. ENc values range from 20 to 61. ENc is a nondirectional measure of codon bias. Higher values suggest equal codon usage, whereas lower values suggest more biased codon usage (Li et al., 2022). ENc was calculated for all 22 transcripts, and average values were calculated for individual gene transcripts. ENc-GC3 was plotted to determine the impact of composition, mutation, and selection forces on codon bias. The data points near or along the curve show the impact of mutational force, whereas the points below the GC3 curve show the impact of selection and other forces (Anwar et al., 2021).

### 2.9. Parity plot analysis

Parity rule 2 (PR2) states that A = T and C = G. Generally, this rule is not precisely followed, thus a deviation is observed. In PR2 bias, the nucleotide skew between A and T and C and G was calculated at the third codon position. A plot was constructed by plotting AT bias (A3/A3 + T3) and GC bias (G3/G3 + C3) on the Y- and X-axes, respectively. If all values are near the center of the plot, A, T, C, and G are used equally (Khandia et al., 2019).

### 2.10. Software used

Scaled Chi-square, CAI, and ENc were calculated using software developed in Bourret et al. (2019). The overall nucleotide composition and the composition at other codon positions were calculated using CAIcal, developed by Puigbò et al. (2008). Graphs and figures were generated, and PCA plots were constructed using Origin18 software. Correlation analysis was performed using Past4.11 software. Despite the low statistical significance, we have to proceed with the available number of transcripts, which is unavoidable because of the inherently low transcript number available for the envisaged genes. Codon frequency and codon pair context were derived using Anaconda 2 software (ANACONDA v.2.0; https://bioinformatics.ua.pt/software/anaconda/).

## 3. Results

### 3.1. Nucleotide composition revealed an elevated prevalence of G in the codon third position

**Figure 1:**
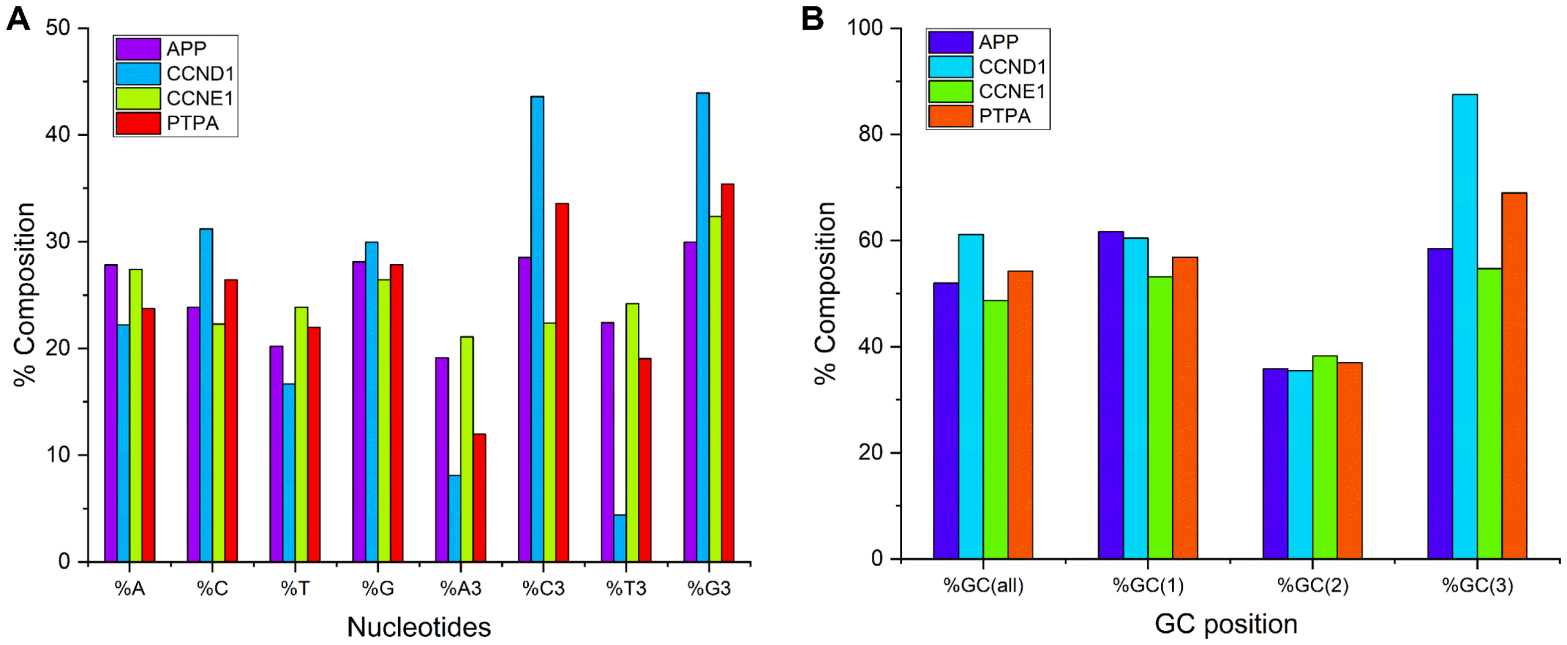
**(A)** Percent nucleotide composition at first and third codon position. **(B)** Percent GC composition at all codon positions.

Studies of gene composition are critical because composition influences several properties including protein stability over a range of temperatures, pH levels, and metal concentrations (Franzo et al., 2021). Biased codon usage is due to the underlying genomic composition. Therefore, certain types of mutations are favored (Chen et al., 2004). Average compositional analysis (Figure 1A) revealed that in the *APP*, *CCND1*, and *PTPA* gene transcripts, the average composition of %G was the highest (28.11, 29.95, and 27.84%, respectively), followed by %C3 (28.5, 45.58, and 33.58%, respectively). The average %T was the lowest (20.19, 16.66%, and 21.99, respectively). For *CCNE1* transcripts, the average composition of nucleotide %A was highest (27.42%), and %C was lowest (22.28%). At the third codon position, for all genes, the average percent composition was highest for %G3 (29.95, 43.91, 32.34, and 35.40% for *APP*, *CCND1*, *CCNE1*, and *PTPA* gene transcripts, respectively) and lowest for %A3 (19.12, 8.10, 21.08, and 11.98% for *APP*, *CCND1*, *CCNE1*, and *PTPA* gene transcripts, respectively). Overall GC percentage ranged from 48.72 to 61.14%. For *APP*, *CCND1*, and *PTPA* gene transcripts, average %GC composition (51.98, 61.14, and 54.28%, respectively) was higher than average AT composition (48.01, 38.85, and 45.71%, respectively). For *CCNE1* transcripts, the %AT composition (51.29%) was higher than the %GC composition (48.72%). Since the GC composition is high in at least three out of four gene transcripts, there is a high chance of having preferred codons ending with C or G nucleotides.

Percent GC3 composition is an indicator of codon bias, and GC3-rich and GC3-poor gene products may represent distinct subcellular locations in the human genome (Shen et al., 2015). A comparison of the average overall GC composition and the composition at the three codon positions for all genes is depicted in Figure 1B. It is evident from this study that the %GC composition was lowest at the second codon position.

### 3.2. Gene length correlates with nucleotide %A composition in all genes

For convenience, we divided all transcripts into two sets. One group contained up-regulated transcripts and the other contained down-regulated transcripts. Gene length affects codon bias and gene expression (Duret and Mouchiroud, 1999; Khandia et al., 2022). We performed correlation analysis between gene length and composition (overall composition, and composition at the third codon position), CAI, SCS, GRAVY, AROMA, PC1, and PC2 (Table 2). In both the up-regulated and down-regulated gene transcripts, we found a significant positive correlation between length, %A composition, and SCS. The transcript lengths of the up-regulated genes were significantly correlated with %G3, %GC1, %GC2, GRAVY, AROMA, and PC1. These analyses revealed that length influences the overall %A composition and codon bias in both gene sets. However, in up-regulated gene transcripts, apart from compositional parameters, length also influences protein properties.

**Table 2:** Correlation analysis of transcript length with compositional parameters, codon bias measures, gene expression, and protein properties.

Up-regulated transcript	%A	%C	%T	%G	%A (3)	%C (3)	%T (3)	%G(3)	%GC (all)
r	0.544	−0.159	−0.397	0.346	0.211	0.071	0.330	−0.717	0.007
p	0.029	0.557	0.128	0.190	0.433	0.793	0.212	0.002	0.978
Significance	*	NS	NS	NS	NS	NS	NS	**	NS
Down-regulated transcript	%A	%C	%T	%G	%A(3)	%C(3)	%T(3)	%G(3)	%GC(all)
r	0.904	−0.215	−0.520	0.294	0.757	−0.610	0.449	−0.363	−0.085
p	0.013	0.683	0.290	0.572	0.081	0.198	0.372	0.480	0.873
Significance	*	NS	NS	NS	NS	NS	NS	NS	NS
Up-regulated transcript	%GC(1)	%GC(2)	%GC(3)	CAI_59	Scaled_Chi	GRAVY	AROMA	PC 1	PC 2
r	0.780	−0.518	−0.284	0.417	0.968	−0.855	−0.579	0.680	0.156
p	0.000	0.040	0.286	0.108	0.000	0.000	0.019	0.004	0.563
Significance	***	*	NS	NS	***	***	*	**	NS
Down-regulated transcript	%GC(1)	%GC(2)	%GC(3)	CAI_59	Scaled_Chi	GRAVY	AROMA	PC 1	PC 2
r	0.414	0.685	−0.760	−0.760	0.995	−0.804	−0.550	0.760	−0.460
p	0.414	0.133	0.079	0.079	0.000	0.054	0.258	0.079	0.359
Significance	NS	NS	NS	NS	***	NS	NS	NS	NS

***p<0.001; **p<0.01; *p<0.05; NS, non-significant.

### 3.3. Gene expression is highest among all genes for *CCND1*

Codon Adaptation Index analysis was performed for all genes. The average CAI values for *APP*, *CCND1*, *CNE2*, and *PTPA* transcripts were 0.788, 0.861, 0.714, and 0.822, respectively. The highest CAI value is for the *CCND1* gene transcript, followed by *PTPA*. The average CAI value for all genes was high, suggesting high expression of all examined genes.

### 3.4. Codon bias is highest in the *CCND1* gene transcript and lowest in *CCNE1* gene transcripts

ENc correlates negatively with codon bias, with high ENc values suggesting low codon bias. The highest possible ENc value, 61, represents equal use of all codons, and the lowest possible value, 20, represents exclusive use of one codon among a set of synonymous codons. Generally, values less than 35 are considered highly biased, whereas values > 50 suggest low bias. The average ENc values for *APP*, *CCND1*, *CCNE1*, and *PTPA* transcripts were 51.55, 33.64, 57.8, and 50., respectively. Hence, overall bias was low, except in *CCND1*, where ENc was below 35 (Wright, 1990; Munjal et al., 2020).

### 3.5. The codon AGG exhibits the highest loading value in both up-regulated and down-regulated gene sets

**Figure 2:**
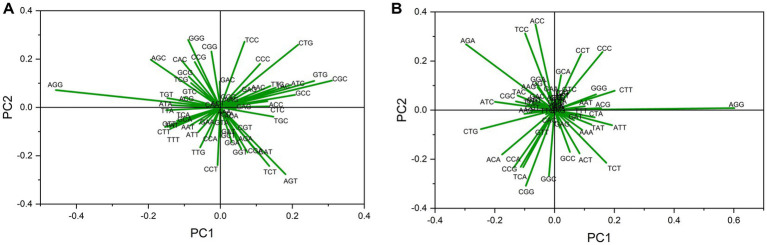
Biplot analysis in PCA in **(A)** up- and **(B)** down-regulated gene transcripts in cancer and neurodegeneration across PC1. Each arrow indicates the loading value of the codon. Codon AGG influencing codon bias the most in both up-regulated and down-regulated gene sets.

Relative synonymous codon usage values were used as descriptor variables in an unsupervised classification method PCA to explore codon usage features. A biplot analysis was performed for both gene sets. The five highest loading values across Axis 1 are listed in Supplementary Table 1. For up-regulated gene sets, 61.51 and 34.72%, and for down-regulated genes, 42.22 and 39.23% contributions to data inertia were attributed to axes 1 and 2, respectively. These results indicate that codon bias influences codon usage patterns. These results suggest that most can be explained by the first two axes (Yu et al., 2021a). High loading values indicate the most influential codons in shaping codon bias (Alqahtani et al., 2022). This analysis revealed lengthy arrows for AGG and CTG codons in both sets (Figures 2A,B), suggesting a strong influence of these codons on codon usage in both gene sets. All other highly influential codons were dissimilar between gene sets.

### 3.6. Relative synonymous codon usage analysis revealed a preference for codons ending in G/C

**Figure 3:**
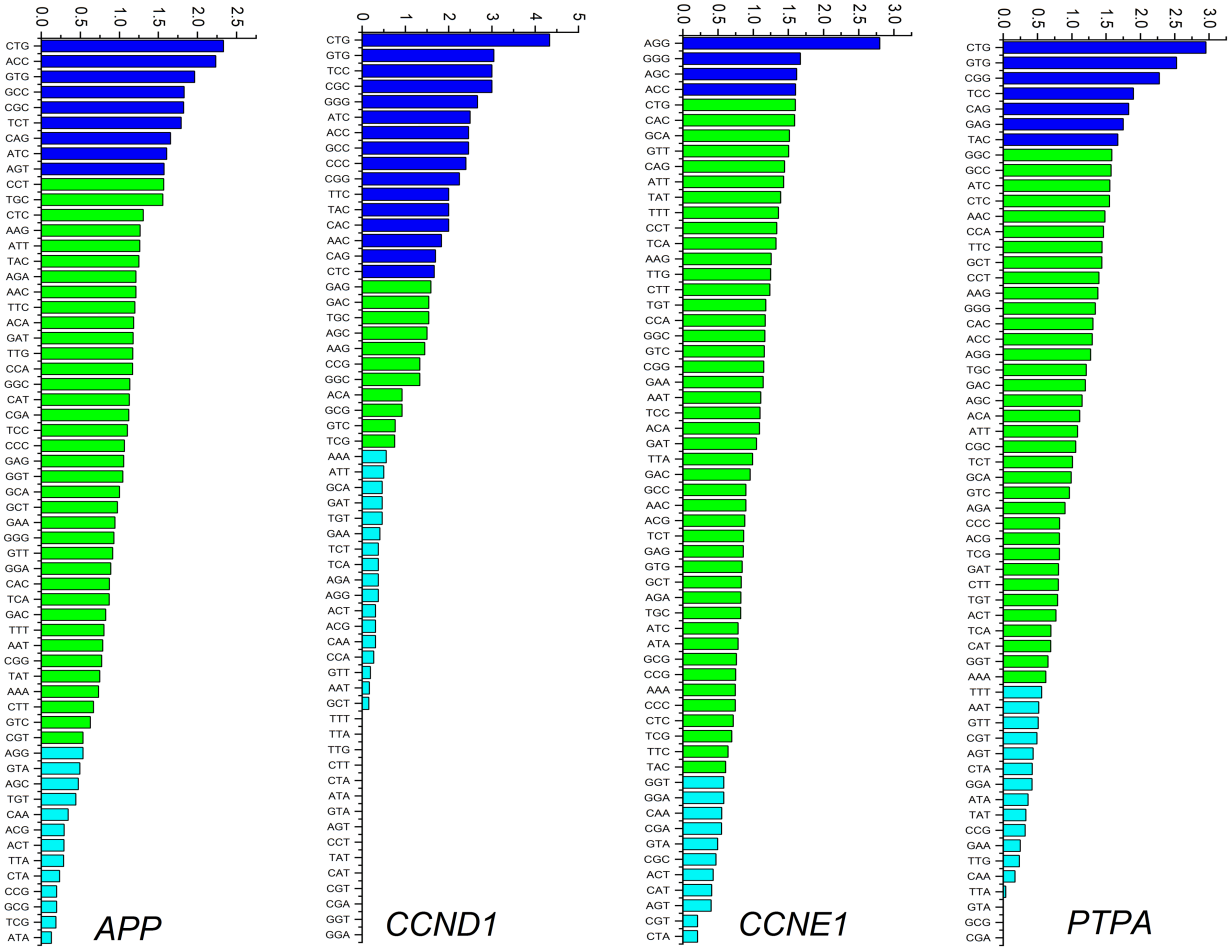
Codon usage analysis for *APP*, *CCND1*, *CCNE1*, and *PTPA* genes. Overexpressed codons (RSCU > 1.6) are depicted as dark blue bars, randomly used codons (RSCU between 1.6 and 0.6) are depicted as green bars, and underrepresented codons are depicted as light blue bars.

Average RSCU analysis of all four gene transcripts revealed that for 16 of 18 amino acids, G/C ending codons were preferred in at least three genes. For the remaining two amino acids, two genes preferred A/T endings and the other two preferred G/C endings. These results suggest an overall preference for codons ending in C. Codon usage for individual genes is shown in Figure 3. Leucine (CTT) and valine (GTT) are the two most frequently used amino acids in all human coronaviruses (Hou, 2020). In the present study, among the genes simultaneously up-regulated or down-regulated in cancer and neurodegeneration, the CTG codon encoding leucine was the most preferred codon for *APP, CCND1*, and *PTPA*, while AGG was the most preferred codon for *CCNE1*. Nine, 16, 4, and 7 codons were overrepresented in *APP*, *CCND1*, *CCNE1*, and *PTPA* gene transcripts, respectively. Similarly, 13, 17, 11, and 14 codons were under-represented in *APP*, *CCND1*, *CCNE1*, and *PTPA* transcripts, respectively. The codons CTA (Leu), GTA (Val), CAA (Gln), and CGT (Arg) were underrepresented in all four genes.

### 3.7. Parity analysis reveals a preference for T and G in codon third positions

**Figure 4:**
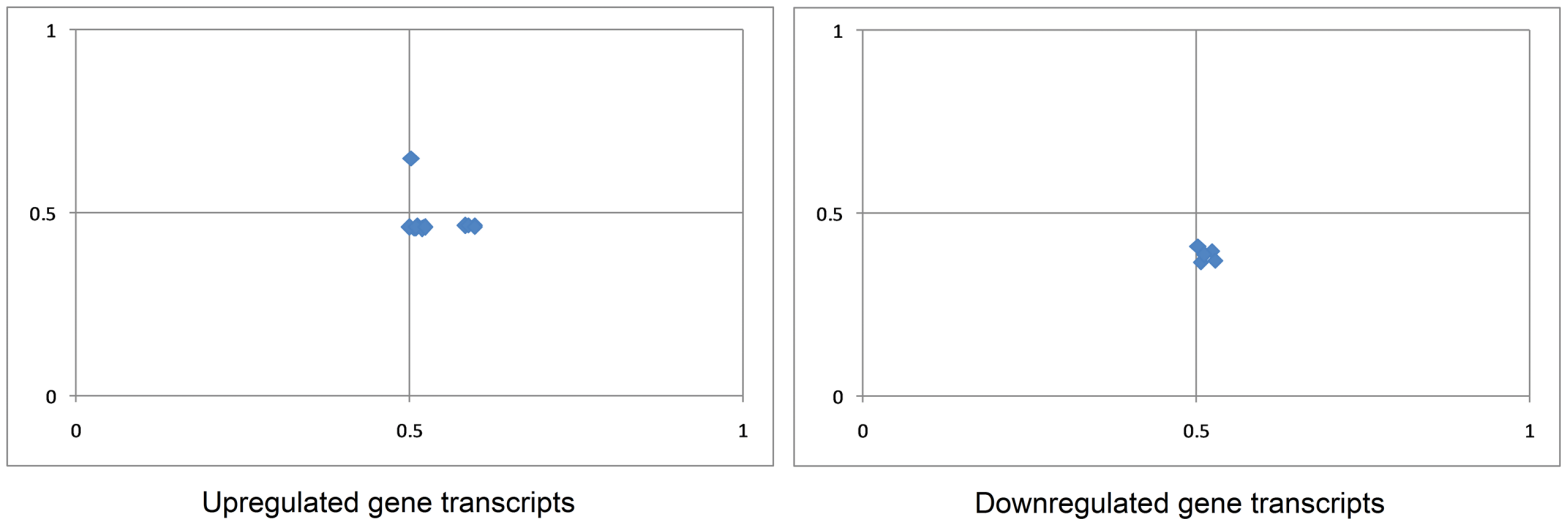
Parity plot analysis of gene transcripts up- and down-regulated in cancer and neurodegeneration revealed that in both sets, A is preferred over T, and C is preferred over G.

At the center of the parity plot, where the value of both coordinates is 0.5, the numbers of A and T nucleotides will be similar, and reciprocal to G and C nucleotides in codon third positions. This is where no selection or mutational force is applied (Sueoka, 1988). In the present study, the mean values of GC and AT bias were 0.531 ± 0.03 and 0.473 ± 0.04 for up-regulated transcripts, and 0.512 ± 0.01 and 0.386 ± 0.02 for down-regulated transcripts. An average bias value of less than 0.5 suggests a preference for pyrimidine over purine (Zhang et al., 2018). Therefore, for both up-regulated and down-regulated gene transcripts, T was preferred over A, and G was preferred over C (Figure 4).

### 3.8. Assessment of selectional, mutational, and compositional constraints in shaping codon bias

**Figure 5:**
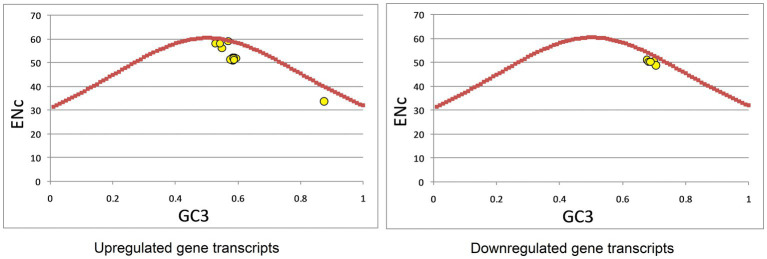
Effective number of codons (ENc)-GC3 analysis of up- and down-regulated genes in cancer and neurodegeneration.

An ENc-GC3 plot was constructed to investigate the forces influencing codon bias. In the presence of data points on the solid curve, codon bias is considered to result from compositional constraints only (Franzo et al., 2021; Khandia et al., 2021), while if data points are present below the expected Nc curve, other forces, such as natural selection, gene length, and RNA structure also influence codon usage (Yu et al., 2021b). Data points near the solid curve indicate the role of mutational forces (Chen et al., 2017). In the up-regulated gene set, data points were present on the %GC3 curve, near the curve, and below the curve, indicating that composition, mutation, and selection forces shape codon usage. In the down-regulated gene set, data points were present near and below the curve, indicating that selection and mutational forces may shape codon usage (Figure 5). To further ascertain the role of mutational forces, we performed a correlation analysis between nucleotide composition and codon composition (A3s, C3s, G3s, U3s, and GC3s), and ENc and codon composition (Supplementary Table 2). Correlation analysis revealed that for the up-regulated gene set, there was a statistically significant correlation between the overall nucleotide and codon composition at the third codon position, except for T-G3 and G-G3. ENc also exhibited a highly significant correlation with codon composition. In contrast, for the down-regulated gene set, only A-A3, T-A3, and ENc-T3 were significantly correlated. These results suggest the role of mutational forces was stronger in up-regulated gene sets than in down-regulated gene sets.

### 3.9. CGT codon was rare in all four genes

Rare codons occur less frequently in a given gene or transcript. At open reading frame 5′ ends, a small cluster of rare codons is generally present that limits the rate of translation to promote effective post-translational folding and prevent ribosome traffic jams (Bentele et al., 2013). Rare codons also influence protein functions (Rosano and Ceccarelli, 2009). Introducing rare codons into a highly expressed gene may reduce the expression levels of that gene and other genes due to reduced availability of the corresponding tRNAs (Frumkin et al., 2018).

**Figure 6:**
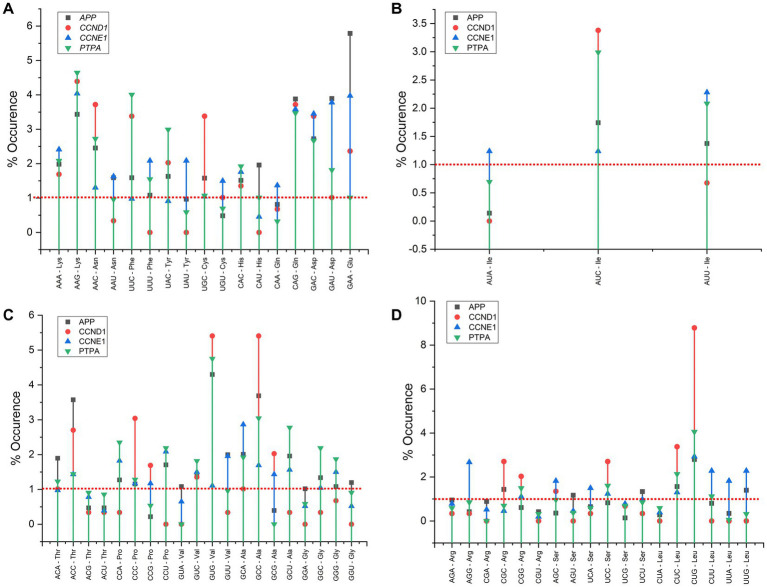
**(A–D)** Average adjusted frequency of the codons in *APP*, *CCND1*, *CCNE1*, and *PTPA* gene transcripts for two-, three-, four- and six-fold degenerate codons. Codons below red dotted lines are rare codons in respective genes. Axis X indicated adjusted occurrence of codons and Axis Y shows respective codons.

Codons with a frequency of < 0.5% in a transcript are considered rare. The adjusted frequencies of the two-, three-, four-, and six-fold degenerate codons are shown in Figure 6. Codons ACG, ACT, AGC, AGG, ATA, CCG, CTA, CGT, GCG, TCG, TTA and TGT codons for APP, AAT, ACG, ACT, AGA, AGG, AGT, ATA, CAT, CCA, CCT, CGA, CGT, CTA, CTT, GCT, GGA, GGC, GGT, GTA, GTT, TAT, TCA, TCT, TTA, TTG, TTT for CCND1; codons ACT, AGT, CAT, CGC, CGT, CTA for CCNE1 gene and codons AGT, CAA, GCA, CGT, GCG, GTG, GTA, TTA, TTG were rare in the PTPA gene. The CGT codon was rare in all four genes, whereas ACT, AGT, CTA, and TTA codons were rare in at least three genes. The ATA, CAT, GCG, GTA, TTG, and CAT codons were rare in at least two genes. Information on rare codon frequencies may help to manipulate multiple genes simultaneously.

### 3.10. High frequency codon pair analysis revealed presence of glutamic acid initiated codon pairs

Three of the four gene transcripts displayed identical codon pairing. Three in the *APP* gene (ACC–ACC, GAA–GAA, and GAG–GAG), two in *CCND1* (GAG–GAG and CTG–CTG), and one in the *PTPA* gene (GCT–GCT). In *APP*, of the 15 highly occurring codon pairs, seven were glutamic acid-initiated, three were aspartic acid-initiated, and two were initiated with valine and alanine codons. In the *CCND1* transcript, alanine and phenylalanine initiate three codon pairs, and leucine and valine initiate two codon pairs. In *CCNE1* transcripts, glutamic acid, leucine, and aspartic acid initiate two codon pairs. In *PTPA*, leucine and glutamic acid initiate three codon pairs, and valine and phenylalanine initiate two codon pairs each. The results suggest that glutamic acid, aspartic acid, leucine, valine, and phenylalanine-initiated codon pairs are abundant in the envisaged genes. The top 15 most frequently occurring codon pairs are listed in Table 3.

**Table 3:** Top 15 high occurring codon pairs in *APP*, *CCND1*, *CCNE1*, and *PTPA* transcripts.

*APP* codon pairs	No of codons	Adjusted score	*CCND1* codon pairs	No of codons	Adjusted score	*CCNE1* codon pairs	No of codons	Adjusted score	*PTPA* codon pairs	No of codons	Adjusted score
ACC–ACC	77	0.98	GAG–GAG	11	3.72	AGG–GAG	13	0.85	CTG–GAG	18	0.96
GAA–GAA	66	0.84	GAG–GTC	3	1.01	GAT–GAA	12	0.78	GAC–TAC	17	0.91
GAG–GAG	55	0.70	CTG–GAG	3	1.01	ATT–GCA	10	0.65	TTC–ATC	16	0.85
GTG–GAA	44	0.56	CTG–CTG	3	1.01	TTG–GAT	8	0.52	GAT–GAG	16	0.85
GCC–AAC	44	0.56	TTC–CTG	2	0.68	TTC–TCG	8	0.52	TTC–AAG	12	0.64
GCA–GAA	44	0.56	TTC–CTC	2	0.68	TTA–ATG	8	0.52	GTG–GAT	12	0.64
GAT–GAG	44	0.56	TTC–ATT	2	0.68	TGT–GTC	8	0.52	GTC–CCT	12	0.64
GAA–GCC	44	0.56	TGC–GAG	2	0.68	TCT–GAA	8	0.52	GCT–GCT	12	0.64
GAA–GAG	39	0.50	TCC–TAC	2	0.68	GGG-AGC	8	0.52	GAT–GAC	12	0.64
GTG–GAG	33	0.42	TCC–ATG	2	0.68	GCC–ATG	8	0.52	GAG–AAG	12	0.64
GAT–GAC	33	0.42	GTG–GCC	2	0.68	GAG–GAA	8	0.52	CTG–CCC	12	0.64
GAG–GAA	33	0.42	GTG–GAC	2	0.68	GAC–AAA	8	0.52	CTC–TGC	12	0.64
GAG–AGA	33	0.42	GCG–GAG	2	0.68	GAA–GAT	8	0.52	CAG–CTG	12	0.64
GAG–ACA	33	0.42	GCC–GCA	2	0.68	GAA–ATG	8	0.52	AAG–TTC	12	0.64
GAC–AAG	33	0.42	GCC–GAG	2	0.68	CTT–CTG	8	0.52	AAG–GCC	12	0.64

**Figure 7:**
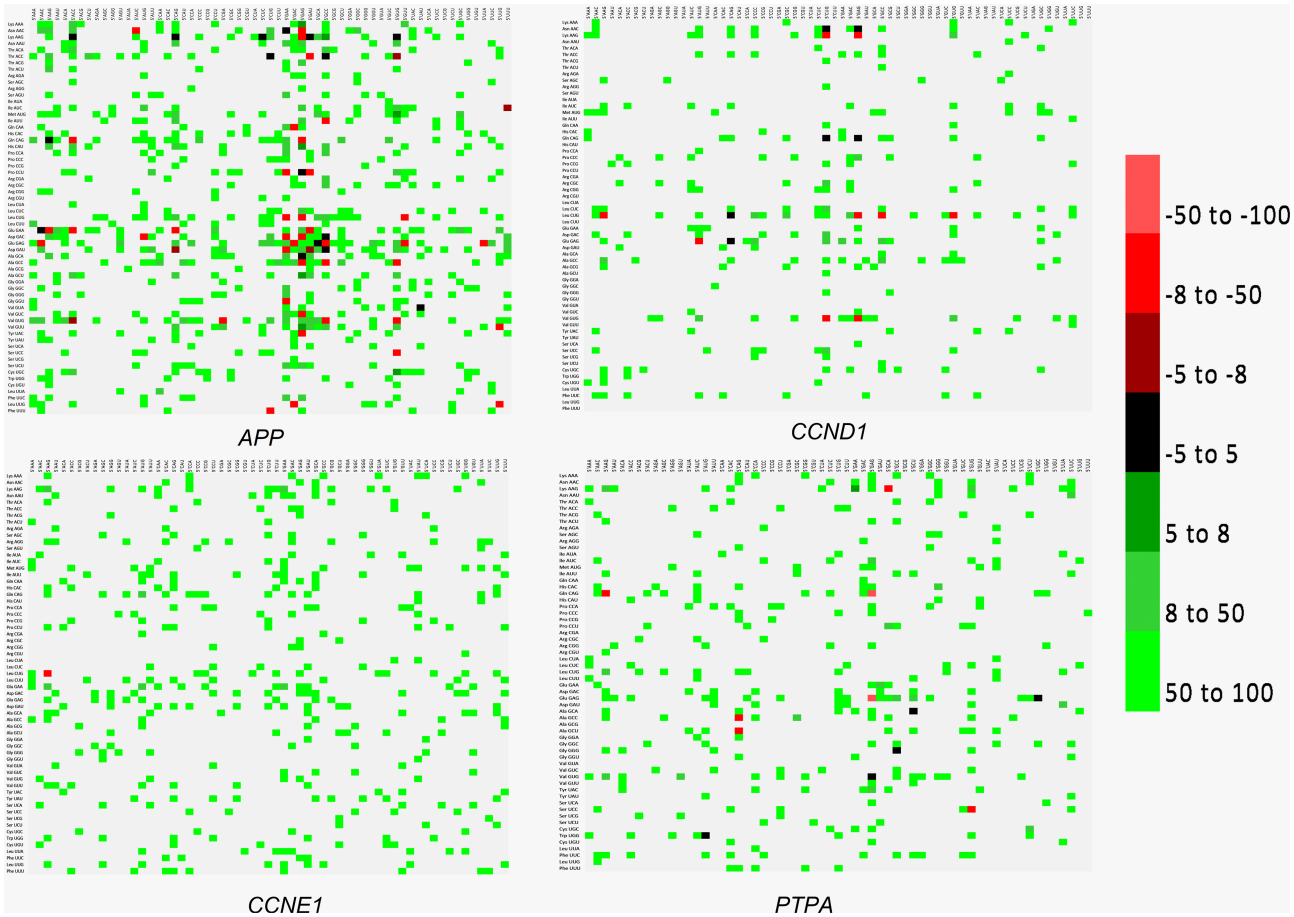
Codon context analysis for *APP*, *CCND1*, *CCNE1*, and *PTPA* genes. Good context (when the 3′ codons appear more frequently than expected) is indicated as positive values (indicated with green), and bad context (3′ codons appear less frequently than expected) is indicated as negative values (Red color). Values between − 5 to + 5 are not statistically significant (no bias and depicted as black color). No correlation is depicted with the grey color.

Codon context bias reveals a preference for the sequentiality of a pair of codons. In addition to the codon pair bias, codon pair context, specifically, context present at the 3′ end has been observed in various organisms, and influences the accuracy and rate of translation. Codon context affects the speed of protein translation and results in translational selection (Tats et al., 2008). Both codon bias and context favor gene expression for a heterologous gene expression (Chung et al., 2013). In the present analysis, in the three transcripts other than *APP*, after the initiating ATG codon, the AAG codon encoding lysine is highly favored (Figure 7).

## 4. Discussion

Cancer and neurodegeneration are ailments with opposite symptoms: cancer is associated with unchecked cellular proliferation, and neurodegeneration is associated with cell death or degeneration. However, the relationships between cancer and neurodegeneration remain incompletely characterized. Patients with Parkinson’s disease, multiple sclerosis, and schizophrenia have lower risk of developing specific cancers (e.g., Parkinson’s disease reduces risk of melanoma, multiple sclerosis reduces risk of brain cancers, and schizophrenia reduces risk of breast cancer; Catalá-López et al., 2014). A few epidemiological studies have revealed that subjects with Alzheimer’s disease (AD) and Parkinson’s disease (PD) have a 35–50% lower risk of cancer. Similarly, cancer patients have lower (35–37%) risk of occurrence of AD and related disorders (Zabłocka et al., 2021). Inverse morbidity results from gene products and genomic pathways being regulated in opposite directions. Many genes and gene products common to both diseases are involved, and mutations in genes such as *PINK1*, *DJ-1*, *LRRK2*, *ATP13A2*, *PLA2G6*, *MAPT*, *CDK5*, and others (Plun-Favreau et al., 2010) result in disease. Apart from mutations that result in gain or loss of function in these genes, some mutations in disease conditions upregulate or downregulate gene expression. Metagenomic analysis revealed the simultaneous upregulation of 74 genes in CNS disorders and downregulation in cancers, and another 19 genes were reported to be concurrently up-regulated in cancers and down-regulated in CNS disorders (Ibáñez et al., 2014). Comparatively fewer genes are up-regulated or down-regulated in both disorders. A literature search revealed four genes that meet this criterion. *APP*, *Cyclin* D, and *Cyclin* E are simultaneously up-regulated in cancer and neurodegeneration, and *PTPA* tended to be down-regulated. We chose these genes to study codon usage and other analyses because manipulation of these genes will offer possible genetic routes to mitigating both disorders together.

Codon usage analysis reveals molecular patterns within a gene or transcript that can influence gene expression (Quax et al., 2015; Zhou et al., 2016). Codon usage is influenced by gene composition (Alqahtani et al., 2021; Simón et al., 2021). Compositional analysis revealed that in the *APP*, *CCND1*, and *PTPA* gene transcripts, %G and %T displayed maximum and minimum respective prevalences. In contrast, in the *CCNE1* transcripts, %A and %C displayed the highest and lowest respective prevalences. Notably, at the third codon position, both G and T nucleotides were preferred in both up-regulated and down-regulated gene transcripts. Therefore, the nucleotide bias at the third codon position is not dependent on composition.

Gene length has been shown to affect gene composition (Alqahtani et al., 2021), codon bias (Duret and Mouchiroud, 1999; Khandia et al., 2022) and gene expression (Duret and Mouchiroud, 1999). We also investigated whether the neurodegeneration- and cancer-related gene transcripts displayed a genuine relationship to these diseases. Gene length was found to correlate with the average frequency of A nucleotides in both the up-regulated and down-regulated transcripts. Furthermore, the %G3, %GC1, and %GC2 components were significantly correlated with the lengths of the up-regulated transcripts. These analyses indicate that only the composition of the up-regulated transcripts is affected by gene length.

Researchers have reported mixed results on the effects of gene length on codon bias. This correlation is strongly positive for *E. coli* genes; strongly negative for *D. melanogaster* and *S. cerevisiae* genes (Moriyama and Powell, 1998), *Caenorhabditis elegans*, and *Arabidopsis thaliana* (Duret and Mouchiroud, 1999); and weak for sesame (Andargie and Congyi, 2022). Codon bias was significantly positively correlated (*p* < 0.001) with gene length in both up-regulated and down-regulated gene sets, indicating that with an increase in length, bias also increased. Gene expression in our study did not correlate with transcript length in either up-regulated or down-regulated genes. Our results differ from those of Brown (2021), who demonstrated that gene expression is inversely proportional to gene length (Brown, 2021).

Because CAI is a significant predictor of expression levels (Park et al., 2012), it has been used as a surrogate marker for expression of several human genes, including *HPRT1* (De Mandal et al., 2020), Tlr7, Tlr9 (Newman et al., 2016), *SPANX* (Choudhury and Chakraborty, 2015), *SRY* (Cai et al., 2015), human oncogenes (Mazumder et al., 2014), and human transcriptome data of monocytes, B, and T lymphocytes (Ruzman et al., 2021). Average CAI values for *APP*, *CCND1*, *CCNE2*, and *PTPA* transcripts were 0.788, 0.861, 0.714, and 0.822, respectively, suggesting a high level of protein expression for all four genes. The highest CAI among all *E. coli* genes was 0.85 for the most abundant LPP protein in *E. coli* cells (Henry and Sharp, 2007). In the dementia-associated gene set, the maximum CAI value found (0.849) was for *CTSD* (Alqahtani et al., 2022). *APP*, *CCND1*, and *CNE2* are associated with cell cycle progression, whereas *PTPA* negatively regulates cell growth and division. Based on the high CAI values of all genes, it is evident that all genes are required for normal cell functioning, and elevated or suppressed expression may lead to disease.

Relative synonymous codon usage analysis revealed that codons ending in GC are favored over codons ending in AT, and 16 of 18 amino acids preferred codons ending in G/C in at least three genes. Our results are in concordance with the results of Newman et al. (2016) based on a study of 19,105 human and 20,558 mouse genes, which revealed that in both species, most of the preferred codons had high GC content. Codons CTA (Leu), GTA (Val), CAA (Gln), and CGT (Arg) were underrepresented in all four genes. When CTA was assessed in Tlr7 and Tlr9, the frequency in Tlr7 was 14.4%, whereas in Tlr9, similar to our study, the frequency was low (0.5%; Newman et al., 2016). In the present study, we found that CTG, which encodes leucine, was the most preferred codon in *APP*, *CCND1*, and *PTPA*, as well as in genes common to primary immunodeficiency and cancer (Khandia et al., 2021). These results suggest that glutamic acid, aspartic acid, leucine, valine, and phenylalanine-initiated codon pairs are abundant in the studied genes.

AGG is the most preferred codon in the *CCNE1* gene, and an AGG cluster near the ORF 5′ end may increase biological activity (Ivanov et al., 1997). This codon is generally rare in *E.coli*. The advantage of the AGG codon is revealed via protein engineering through reassignment of the AGG sense codon using an orthogonal tRNA CCU and an aminoacyl-tRNA synthetase pair resulting in charging of the tRNA with an unnatural or chemically modified amino acid. The abundance of the AGG codon in *CCNE1* could thus be exploited for protein engineering to interrogate other physiological functions (Lee et al., 2015). While recording the genetic sequences of our selected genes to manipulate gene expression profiling, it must be kept in mind that when AGG and TTG codon frequencies increase, the frequencies of other C- or G-ending codons decrease, negatively influencing gene expression in humans. Local compositional biases may not explain this unusual behavior (Kliman and Bernal, 2005).

Rare codons such as AGG, AGA, CUA, AUA, CGA, and CCC have been used to fine tune gene expression in *E. coli* (Wang et al., 2016). A cluster of rare codons present at the 5′ end of the transcript ensures proper protein folding and biological activity (Rosano and Ceccarelli, 2009; Bentele et al., 2013). In this study, CGT codons were rare in all four genes, whereas ACT, AGT, CTA, and TTA codons were rare in at least three genes. In humans, the six codons, GCG (Ala), CCG (Pro), CGT (Arg), CGC (Arg), TCG (Ser), and ACG (Thr) are rare (Kanduc, 2017). It is thus clear that CGT codons are rarely used in the studied transcripts. However, the low occurrence of other codons may be a result of different negative selections for local pauses in translation that can be beneficial for protein biogenesis (Clarke and Clark, 2008). Sequences optimized with codon-pair context exhibited higher protein expression than the native codons.

The extent to which a codon is translated depends on neighboring codons. This is called a context effect, and influences translation kinetics (Chevance et al., 2014). Sequences optimized using a codon-pair context showed better protein expression than those optimized using codon usage (Huang et al., 2021). Removing only two codon pairs that are detrimental to protein expression may increase protein expression levels 30 fold compared to the original sequence (Trinh et al., 2004). Deoptimized codon pairs have been used to generate attenuated vaccine candidates against influenza, polioviruses, and arboviruses (Jack et al., 2017). The same strategy may be adopted to augment the expression profile to the desired level through gene editing. In the present study, an abundance of glutamic acid-, aspartic acid-, leucine-, valine-, and phenylalanine-initiated codon pairs were observed, and disruption of preferred codon pairs can be used to reduce the gene expression level (Jack et al., 2017). After the ATG codon, a highly positive context was present for the AAG (lysine) codon in all transcripts, except for APP, reflecting a prominent 3′ context effect (Tats et al., 2008). With the help of new scientific developments, it is now possible to replace a copy of a defective gene with the desired gene. This strategy may augment expression levels, raising risk of cancer and/or neurodegeneration.

## 5. Conclusion

From our analysis, it was evident that codons ending in G/C were preferred over codons ending in AT in all genes and such pattern is not the result of nucleotide compositional bias. In the present study, CTA (Leu), GTA (Val), CAA (Gln), and CGT (Arg) were under-represented in all four genes. In contrast, ACT, AGT, CTA, and TTA codons were rare in at least three genes. This information is helpful for reducing gene expression levels by inserting these codons during gene coding to ameliorate disease symptoms. Negative selection of codons is suggestive of specific requirements for local pauses during protein translation. Glutamic acid-, aspartic acid-, leucine-, valine-, and phenylalanine-initiated codon pairs were abundant. Also, the 3′ context of the AAG codon with ATG at the 5′ end was evident. Present study has unavoidable limitation of using four genes *APP*, *CCND1*, *CCNE1*, and *PTPA* only, since so far only four genes have been identified those are commonly implicated in cancer and neurodegeneration. With more number of genes, statistical analyses would be stronger. In the present study, different information gained regarding molecular patterns, codon usage, codon usage bias, nucleotide bias at the third codon position, preferred codons, preferred codon pairs, rare codons, and codon context will guide future studies. Based on this knowledge, these genes may be manipulated to augment their defects through gene editing, CRISPR/Cas, or any other gene augmentation technique.

## Data availability statement

The original contributions presented in the study are included in the article/Supplementary material, further inquiries can be directed to the corresponding authors.

## Author contributions

RK: conceptualization, analysis, software, data curation, writing—review and editing, supervision, project administration, and final approval of the version to be published. MP, SA-H, and IB: conceptualization, data analysis, interpretation of data, revision, critical analysis, and editing. MZ: design of work, software, validation, resources, supervision, project administration, funding acquisition, and intellectual content. PG: conceptualization, analysis, software, data curation, writing—review and editing, supervision, and project administration. All authors contributed to the article and approved the submitted version.

## Conflict of interest

The authors declare that the research was conducted in the absence of any commercial or financial relationships that could be construed as a potential conflict of interest.

## Publisher’s note

All claims expressed in this article are solely those of the authors and do not necessarily represent those of their affiliated organizations, or those of the publisher, the editors and the reviewers. Any product that may be evaluated in this article, or claim that may be made by its manufacturer, is not guaranteed or endorsed by the publisher.

## Footnotes

^1^ http://www.ncbi.nlm.nih.gov
