# Supplementary material for: Application of codon usage and context analysis in genes up- or down-regulated in neurodegeneration and cancer to combat comorbidities
Source: Front Mol Neurosci. 2023 Jun 13;16:1200523. doi: 10.3389/fnmol.2023.1200523 (PMC10293642; doi:10.3389/fnmol.2023.1200523)
Supplement: Supplementary file 1 [file Table_1.DOCX]

Table S1. Top 5 loading values for up and downregualted gene transcripts

| S.No. | Upregulated | | S.No. | Downregulated | |
| --- | --- | --- | --- | --- | --- |
| 1 | AGG | -0.457 | 1 | AGG | 0.602 |
| 2 | CGC | 0.310 | 2 | AGA | -0.296 |
| 3 | GTG | 0.261 | 3 | CTG | -0.246 |
| 4 | CTG | 0.216 | 4 | CTT | 0.201 |
| 5 | GCC | 0.207 | 5 | ATC | -0.200 |

Table S2 Correlation analysis between ENc and nucleotide composition at the third codon position to evaluate the effect of mutational forces

|  | Upregulated Gene transcripts | | | | |
| --- | --- | --- | --- | --- | --- |
|  | %A3 | %C3 | %T3 | %G3 | %GC3 |
| %A | 0.898*** | -0.749*** | 0.934*** | -0.949*** | -0.922*** |
| %C | -0.990*** | 0.963*** | -0.975*** | 0.782*** | 0.983*** |
| %T | 0.780*** | -0.909*** | 0.701** | -0.317^NS^ | -0.734** |
| %G | -0.789*** | 0.890*** | -0.714** | 0.368 ^NS^ | 0.745*** |
| %GC | -0.970*** | 0.985*** | -0.933*** | 0.678** | 0.950*** |
| ENc | 0.956*** | -0.982*** | 0.926*** | -0.661*** | -0.939*** |
|  | Downregulated gene transcripts | | | | |
|  | %A3 | %C3 | %T3 | %G3 | %GC3 |
| %A | 0.865* | -0.372 ^NS^ | 0.097 ^NS^ | -0.467 ^NS^ | -0.527 ^NS^ |
| %C | 0.348^NS^ | 0.003 ^NS^ | -0.329 ^NS^ | 0.337 ^NS^ | 0.091^NS^ |
| %T | -0.933** | 0.339 ^NS^ | 0.152 ^NS^ | -0.052 ^NS^ | 0.356^NS^ |
| %G | 0.106^NS^ | -0.212 ^NS^ | 0.194^NS^ | 0.063 ^NS^ | -0.214 ^NS^ |
| ENc | 0.155^NS^ | -0.658^NS^ | 0.819* | -0.141^NS^ | -0.753^NS^ |

p<0.001***, p<0.01**, p<0.05*, NS Non significant
